# An Acute Stress Scale for Health Care Professionals Caring for Patients With COVID-19: Validation Study

**DOI:** 10.2196/27107

**Published:** 2021-03-09

**Authors:** Jose Joaquin Mira, Angel Cobos, Olga Martínez García, María José Bueno Domínguez, María Pilar Astier-Peña, Pastora Pérez Pérez, Irene Carrillo, Mercedes Guilabert, Virtudes Perez-Jover, Cesar Fernandez, María Asuncion Vicente, Matilde Lahera-Martin, Carmen Silvestre Busto, Susana Lorenzo Martínez, Ascension Sanchez Martinez, Jimmy Martin-Delgado, Aurora Mula, Barbara Marco-Gomez, Cristina Abad Bouzan, Carlos Aibar-Remon, Jesus Aranaz-Andres, SARS-CoV-2 Second Victims Working Group

**Affiliations:** 1 Atenea Research Group Foundation for the Promotion of Health and Biomedical Research Alicante Spain; 2 Department of Health Psychology Miguel Hernandez University Elche Spain; 3 European Research Network on Second Victims (COST Action CA-19113) Alicante Spain; 4 Intensive Care Unit San Cecilio Clinical University Hospital Granada Spain; 5 Psychiatry Department San Cecilio Clinical University Hospital Granada Spain; 6 Department of Patient Safety Sagessa Group Reus Spain; 7 Centro de Salud la Jota Zaragoza Spain; 8 Spanish Society for Quality Care Oviedo Spain; 9 Telematics Engineering Area Miguel Hernandez University Elche Spain; 10 Department of Occupational Health Osasunbidea Pamplona Spain; 11 Patient Safety Department Osasunbidea Pamplona Spain; 12 Department of Quality and Patient Management Alcorcon Foundation University Hospital Madrid Spain; 13 Department of Quality of Care General University Hospital Reina Sofía Murcia Spain; 14 Mental Health Department Calatayud Health Area Zaragoza Spain; 15 Department of Preventive Medicine Lozano Blesa Univerisity Clinical Hospital Zaragoza Spain; 16 Department of Preventive Medicine Ramón y Cajal University Hospital Madrid Spain; 17 Be+ Against COVID Alicante Spain

**Keywords:** SARS-CoV-2 virus, COVID-19 outbreak, medical staff, acute stress, moral injury, posttraumatic stress, COVID-19

## Abstract

**Background:**

The COVID-19 pandemic has affected the response capacity of the health care workforce, and health care professionals have been experiencing acute stress reactions since the beginning of the pandemic. In Spain, the first wave was particularly severe among the population and health care professionals, many of whom were infected. These professionals required initial psychological supports that were gradual and in line with their conditions.

**Objective:**

In the early days of the pandemic in Spain (March 2020), this study aimed to design and validate a scale to measure acute stress experienced by the health care workforce during the care of patients with COVID-19: the Self-applied Acute Stress Scale (EASE).

**Methods:**

Item development, scale development, and scale evaluation were considered. Qualitative research was conducted to produce the initial pool of items, assure their legibility, and assess the validity of the content. Internal consistency was calculated using Cronbach α and McDonald ω. Confirmatory factor analysis and the Mann-Whitney-Wilcoxon test were used to assess construct validity. Linear regression was applied to assess criterion validity. Back-translation methodology was used to translate the scale into Portuguese and English.

**Results:**

A total of 228 health professionals from the Spanish public health system responded to the 10 items of the EASE scale. Internal consistency was .87 (McDonald ω). Goodness-of-fit indices confirmed a two-factor structure, explaining 55% of the variance. As expected, the highest level of stress was found among professionals working in health services where a higher number of deaths from COVID-19 occurred (*P*<.05).

**Conclusions:**

The EASE scale was shown to have adequate metric properties regarding consistency and construct validity. The EASE scale could be used to determine the levels of acute stress among the health care workforce in order to give them proportional support according to their needs during emergency conditions, such as the COVID-19 pandemic.

## Introduction

From the beginning of the COVID-19 pandemic, the rate of reproduction, lethality, and social alarm that accompanies SARS-CoV-2 [1,2] poses a challenge to health systems in all countries. The impact of the COVID-19 pandemic has been devastating for infected and uninfected patients and societies, as well as for the health care workforces who have been considered second victims of SARS-CoV-2.

Although we have more information about the new coronavirus and health institutions are now more capable of responding to patients’ needs, this was not the case at first. At that time, in addition to the initial clinical uncertainty, the shortage of equipment, and the difficulties in maintaining the supply chain, there were constant changes in instructions, the interruption of all nondelayable care, and the experience of isolation of admitted patients, who died alone in more cases than could be admitted. At that time, there was a perception that the uncertainty about COVID-19 patients’ evolution, care pressure, and lack of means affected the emotional balance of health professionals. The most observed responses were related to acute stress, moral injury, and compassion fatigue [3-6].

The Spanish health system, in particular, has been overwhelmed by the number of patients with COVID-19; during the first wave of the outbreak, as of May 26, 2020, there had been 236,259 cases and 27,117 deaths. In addition, 20.4% of COVID-19 patients were health professionals [7]. At that time, the physical and mental effort involved in caring for patients with COVID-19 has caused acute stress, compassion fatigue, and other affective pathologies, which, together with psychosomatic reactions, have affected work morale [4,8,9]. Integrated care has been jeopardized. Health care professionals have been reassigned to areas where they have no expertise or preparation, protocols have been made overnight and are continuously changing, and continuity of care was interrupted at all levels. Under these conditions, health care has been compromised [10]. Moreover, a significant proportion of these professionals were at risk, since most were reluctant to seek help to face affective and anxiety disorders [11].

In these circumstances, the priority was to offer supporting tools to frontline health care providers, including resources they could access to support them in their feelings of being overwhelmed and down. These resources had to be a quick response to the needs identified in informal conversations and in the proposals and experience of leading professionals in organizations who demanded support to deal with everyday stressful situations. Within this framework, the project *Be+ against COVID* emerged, given a reluctance by health care providers to request support; the idea was to develop a self-assessment tool to measure acute stress levels adjusted to the experiences that the professionals described having lived.

The aim of this study was to show the design and validation process of a self-applied acute stress scale for people who worked in the direct care of patients with COVID-19 during the early period of the outbreak; this scale is called the Self-applied Acute Stress Scale (EASE). This instrument focused on facilitating awareness of the level of stress endured by health care professionals; the instrument would then be used to assess the impact of organizational changes in health care systems on coping with the acute stress caused by (1) the limited resources available to treat patients during the early period of this pandemic, (2) uncertainty about the appropriate treatment of COVID-19 patients, (3) the risk of becoming infected in the course of care, and (4) the interrupted care for non-COVID-19 patients. As a result, this could contribute to secondary prevention of emotional and anxiety responses once the critical phase of the pandemic is over.

## Methods

### Overview

**Figure 1:**
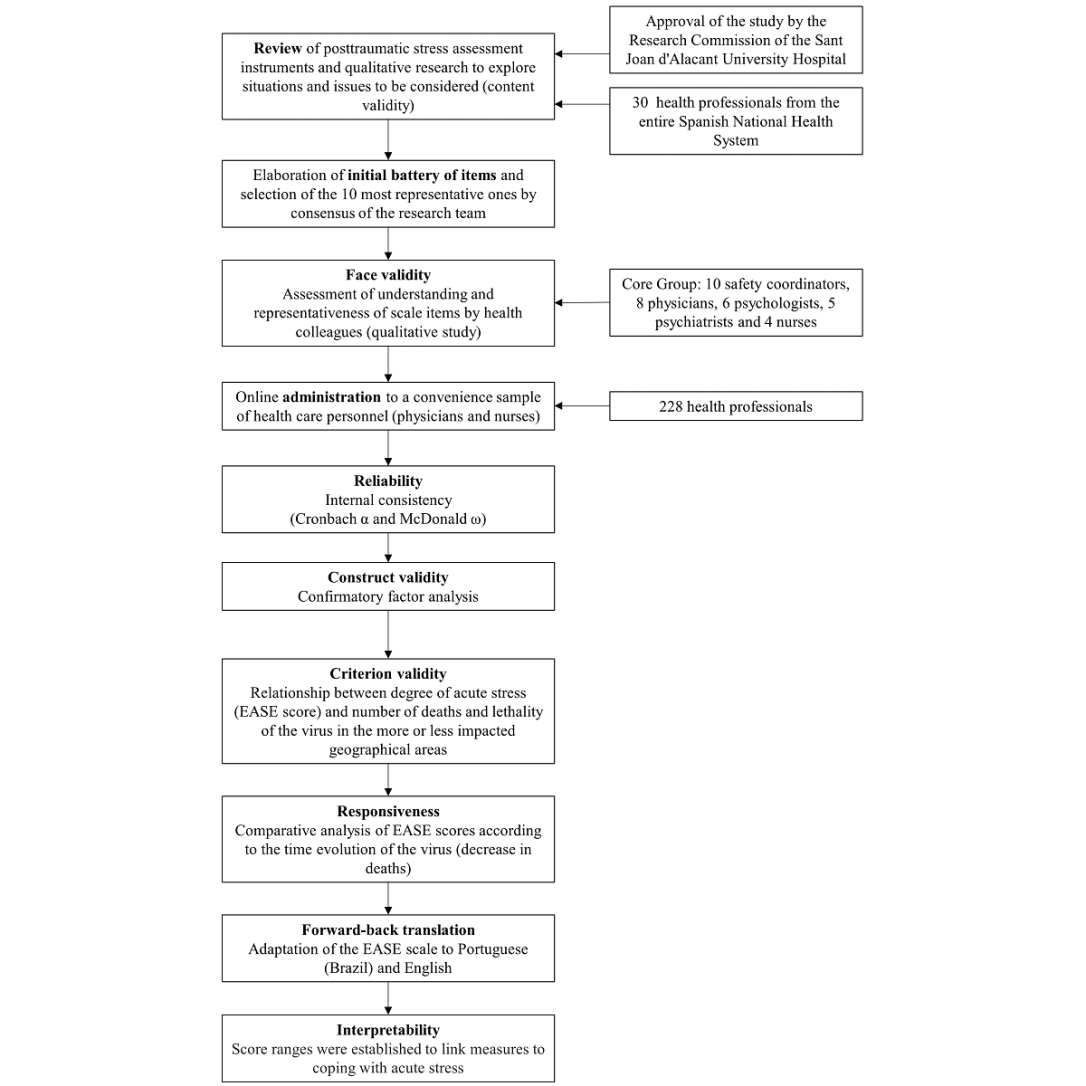
Flow diagram of the Self-applied Acute Stress Scale (EASE) validation process.

This study was conducted in Spain between March 20 and April 19, 2020, and coincided with the worst moments of the first wave of the COVID-19 outbreak. Health care providers could complete the EASE scale on a website or via a mobile app (see Figure 1). This study’s protocol considered the three phases to creating a scale that were described by Boateng et al (ie, item development, scale development, and scale evaluation) [12], the standards and guidelines for validation practices summarized by Chan [13], and the COnsensus-based Standards for the selection of health status Measurement INstruments (COSMIN) recommendations [14]. This study was approved by the Research Commission of the Sant Joan d'Alacant University Hospital.

The target population of this instrument was the health care workforce, which includes physicians, nurses, and other health care personnel. The rationale for developing a new instrument was based on the demanding circumstances that were being experienced during the pandemic. Another reason was to develop a short tool, to be answered in less than 5 minutes, that identified the problematic situations being experienced during the early period of the pandemic by health care professionals to motivate them to look for support in facing the affective- and anxiety-related situations described. Other instruments, such as general scales for posttraumatic stress disorder, anxiety, and depression, were discarded due to their focus on mental health and because they did not include items reflecting the experiences that were observed to be more frequent during the treatment of COVID-19 patients [12,13].

### Definitions

Acute stress was defined as an intense, unpleasant, and dysfunctional reaction beginning shortly after an overwhelming traumatic event and lasting less than a month due to a particularly stressful event. Acute stress responses may be adaptive but, in other cases, may impact well-being over time [15].

### Participants

A convenience sample of physicians, nurses, and other health care personnel were recruited. The sample size was adjusted according to the number of items (ie, maximum of 10), applying the criterion of a minimum of 20 subjects with valid responses per item. To preserve complete anonymity, no information on age or experience in the workplace was requested.

### Content Validity

A pragmatic literature review of empirical articles, letters, and reviews describing the experiences of the health care workforce was conducted. Additionally, eight physicians, four nurses, five psychiatrists, six psychologists, and 10 safety personnel from 10 hospitals and four health centers (Core Group) participated in collecting information during informal interviews with their colleagues. All of this information was grouped into categories according to the similarity of problematic situations. In this way, the sets of problematic situations and experiences were mapped and grouped into two theoretical factors: affective responses and anxiety responses (ie, content validity).

### Item Generation

Two to three items were developed for each problem situation by the research team considering the results of the literature review—regarding previous SARS, Middle East respiratory syndrome (MERS), and Ebola outbreaks and studies in progress on the impact of the COVID-19 outbreak—and the emotional experiences and signs of acute stress reported by professionals in health care centers where the team members were working. Items measuring acute stress in health care professionals were assessed for possible inclusion. The successive approximations method was applied. In successive consensus rounds, the items considered most appropriate were either discarded or selected.

### Face Validity and Legibility

The resulting scale was assessed by the Core Group considering its representativeness (ie, face validity). The Core Group also assessed the understanding of each item (ie, legibility) and the response options on a 4-point scale.

### Reliability

Internal consistency was calculated using Cronbach α and McDonald ω. A value greater than .70 was considered acceptable for this analysis.

### Construct Validity

Confirmatory factor analysis (CFA) was used to confirm the underlying two-factor structure, estimating several fit indices to test which CFA model best represented the data set: the comparative fix index, Jöreskog and Sörbom’s adjusted goodness-of-fit index, the standardized root mean square residual, Jöreskog and Sörbom’s goodness-of-fit index, and the normed fit index. Additionally, we tested the hypothesis stating that scores on the EASE scale would be higher for those professionals working in centers located in territories with higher mortality from COVID-19. Using the Mann-Whitney-Wilcoxon test and linear regression analysis (ie, the *Enter* method), the capacity to classify the professionals’ responses in the EASE scale was assessed according to the number of deaths registered in the geographical area where the health center was located during the period from March 10 to April 19, 2020.

### Translatability

The translation-back-translation method was used to ensure language and cultural equivalence between the Portuguese and English versions of the scale.

### Criterion Validity

Criterion validity was determined by the scale’s ability to discriminate between levels of acute stress over time, hypothesizing that it would be greater in those cases with higher care burden. We compared the EASE scale scores during two different periods of the pandemic’s evolution—March 25 to April 1, 2020, and April 14 to 19, 2020—which were marked by different daily numbers of cases and deaths by COVID-19 (ie, between 800 and 900 deaths/day versus <500 deaths/day, respectively). The data published by the Ministry of Health on April 22, 2020, were taken as a reference.

### Interpretability

To determine the impact on well-being, a consensus among researchers was established to understand scores using four segments: scores up to the 50th percentile, scores between the 50th and 80th percentile, scores between the 80th and 90th percentile, and scores above the 90th percentile.

## Results

### Overview

The first 228 responses from health care professionals were included in the database and used to evaluate the scale. A total of 42.1% (96/228) of respondents were physicians, 28.1% (64/228) were nurses, and 29.8% (68/228) were health support staff (ie, nursing assistants and care attendants). They mostly worked in Madrid (66/228, 28.9%), Andalusia (50/228, 21.9%), Valencia (40/228, 17.5%), and Catalonia (15/228, 6.6%). All key groups were adequately represented. The subsample of physicians was slightly overrepresented considering the proportion of physicians on staff to nurses. The responses originated from both territories—one with the highest and one with the lowest incidence of COVID-19—proportional to where the incidence of new cases of COVID-19 was high and moderate.

### Content Validity, Face Validity, and Legibility

The most relevant sources of acute stress identified from the professionals’ experiences were constant changes in instructions, shortage of material to avoid contagion, reduction in the number of staff due to the risk exposure or contagion, bitter feelings when seeing patients die lonely, fear of infecting their families, making decisions reserved for situations of major catastrophes with a high component of ethical conflict, and the passing away of colleagues. These issues were represented by an initial set of 17 possible items on the EASE scale. This number was finally reduced to 10 items (ie, version 0 of the scale) once participants considered their representativeness and comprehension (see Multimedia Appendix 1).

### Reliability

Internal consistency values were adequate—Cronbach α=.85 and McDonald ω=.87—when all items of the EASE scale were considered. For the affective response factor, Cronbach α=.81 and McDonald ω=.81; for the fear and anxiety factor, Cronbach α=.73 and McDonald ω=.74.

### Construct Validity

**Figure 2:**
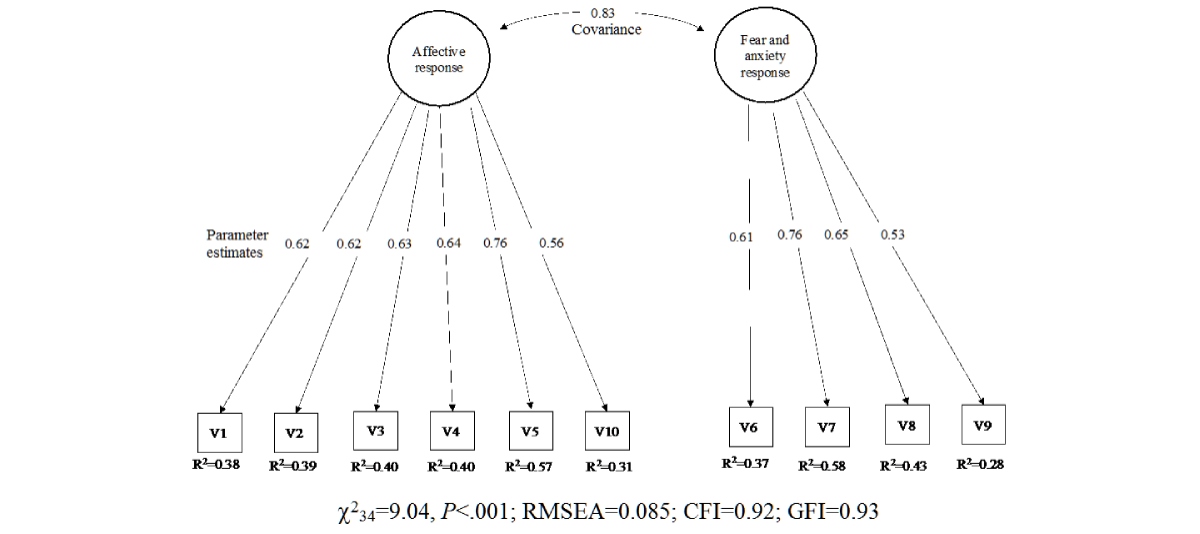
Path diagram of the confirmatory factor analysis. Standardized weights and measurement errors of each item of the Self-applied Acute Stress Scale (EASE). CFI: comparative fix index; GFI: goodness-of-fit index; RMSEA: root mean square error of approximation; V1: I can’t help but think of recent critical situations. I can't get out of work; V2: I have completely lost the taste for things that gave me peace of mind; V3: I keep my distance, I resent dealing with people, and I'm irascible even at home; V4: I feel that I am neglecting many people who need my help; V5: I have difficulty thinking and making decisions, I have many doubts, and I have entered a kind of emotional blockage; V6: I feel intense physiological reactions (shock, sweating, dizziness, shortness of breath, insomnia, etc) related to the current crisis; V7: I feel on permanent alert. I believe that my reactions now put other patients, my colleagues, or myself at risk; V8: Worrying about not getting sick causes me a strain that is hard to bear; V9: I'm afraid I'm going to infect my family; V10: I have difficulty empathizing with patients' suffering or connecting with their situation (emotional distancing and emotional anesthesia).

The CFA indicated an acceptable fit to the data (see Table 1 and Figure 2)*.* Two factors explaining 55% of the variance were confirmed by CFA. The first factor measured affective responses and the second factor measured fear and anxiety responses. Global scores ranged from 3 to 30 (see Table 2). They were higher among professionals working in health services that accumulated a higher number of deaths (mean 10.6, 95% CI 9.5-11.7 vs mean 8.2, 95% CI 6.5-9.9; *P*<.05). This tendency was also observed in the scores for the two-solution factors (affective responses: mean 6.3, 95% CI 5.6-7.9 vs mean 4.9, 95% CI 3.7-6.1; fear and anxiety responses: mean 4.3, 95% CI 2.8-4.8 vs mean 3.2, 95% CI 2.4-4.0). Lethality rate was positively related to the EASE scale scores (β=1.07, 95% CI 1.00-1.15; *P*<.05) (see Table 3).

**Table 1:** Confirmatory factor analysis to determine fit to the data.

Goodness-of-fit index		Value
**Absolute adjustment rates**		
	Standardized root mean square residual	0.06
	Jöreskog and Sörbom’s goodness-of-fit index	0.92
	Jöreskog and Sörbom’s adjusted goodness-of-fit index	0.90
**Relative adjustment rates**		
	Normed fit index	0.90
	Comparative fit index	0.93

**Table 2:** Construct validity of the acute stress scale used among professionals caring for patients with COVID-19.

Scale item		Scale score (N=228), mean (SD), 95% CI^a^	Loading
**Factor 1: affective responses**			
	I keep my distance, I resent dealing with people, and I am irascible even at home.	1.1 (0.9), 1.0-1.2	0.83
	I have completely lost the taste for things that used to bring me peace of mind or well-being.	1.1 (0.9), 1.0-1.2	0.72
	I feel that I am neglecting many people who need my help.	0.9 (0.9), 0.8-1.0	0.71
	I cannot help but think of recent critical situations. I can’t get out of work.	1.3 (0.1), 1.2-1.4	0.60
	I have difficulty thinking and making decisions, I have many doubts, and I have entered a kind of emotional blockage.	0.9 (0.9), 0.8-1.0	0.58
	All factor 1 items	6.0 (3.9), 5.5-6.5	N/A^b^
**Factor 2: fear and anxiety responses**			
	I have difficulty empathizing with patients’ suffering or connecting with their situation (emotional distancing and emotional anesthesia).	0.7 (0.9), 0.6-0.8	0.48
	Worrying about not getting sick causes me a strain that is hard to bear.	0.8 (0.9), 0.7-0.9	0.86
	I’m afraid I’m going to infect my family.	1.3 (1.0), 1.2-1.4	0.70
	I feel on permanent alert. I believe that my reactions now put other patients, my colleagues, or myself at risk.	0.9 (0.9), 0.8-1.0	0.66
	I feel intense physiological reactions (shock, sweating, dizziness, shortness of breath, insomnia, etc) related to the current crisis.	1.0 (0.9), 0.9-1.1	0.55
	All factor 2 items	4.0 (2.8), 3.6-4.4	N/A
Total (score ranges from 3 to 30)		10.0 (6.1), 9.2-10.8	N/A

^a^Individual scores range from 0 to 3.

^b^N/A: not applicable; this value was calculated for individual items only.

**Table 3:** Acute stress scale used among professionals caring for patients with COVID-19 over two periods and two geographical areas.

Scale item	Scale score (N=228), mean (SD), 95% CI^a^			
	First period: 800-900 deaths per day^b^	Second period: <500 deaths per day^b^	Geographical area: more impact^c^	Geographical area: less impact^d^
I can’t help but think of recent critical situations. I can’t get out of work.	1.5 (0.5), 1.2-1.8	0.8 (1.0), 0.4-1.2	1.4 (1.0), 1.2-1.6	1.2 (0.9), 0.9-1.5
I have completely lost the taste for things that gave me peace of mind.	1.5 (0.5), 1.2-1.8	1.2 (1.0), 0.8-1.6	*1.2 (0.9), 1.1-1.3*	*0.8 (0.8), 0.5-1.1*
I keep my distance, I resent dealing with people, and I'm irascible even at home.	*1.6 (0.9), 1.0-2.2*	*0.8 (0.8), 0.4-1.2*	1.2 (1.0), 1.0-1.4	0.9 (0.8), 0.6-1.2
I feel that I am neglecting many people who need my help.	1.3 (0.9), 0.7-1.9	0.8 (1.0), 0.4-1.2	0.9 (0.9), 0.8-1.0	0.8 (0.9), 0.5-1.1
I have difficulty thinking and making decisions, I have many doubts, and I have entered a kind of emotional blockage.	*1.5 (0.9), 0.9-2.1*	*0.6 (0.8), 0.2-1.0*	1.0 (1.0), 0.8-1.2	0.7 (0.9), 0.4-1.0
I feel intense physiological reactions (shock, sweating, dizziness, shortness of breath, insomnia, etc) related to the current crisis.	1.5 (0.8), 0.9-2.1	1.1 (0.9), 0.7-1.5	1.1 (1.0), 0.9-1.3	0.8 (0.9), 0.5-1.1
I feel on permanent alert. I believe that my reactions now put other patients, my colleagues, or myself at risk.	1.6 (1.1), 0.8-2.4	0.8 (0.8), 0.4-1.2	0.9 (1.0), 0.7-1.1	0.6 (0.7), 0.4-0.8
Worrying about not getting sick causes me a strain that is hard to bear.	1.1 (1.0), 0.4-1.8	0.7 (0.9), 0.3-1.1	0.9 (0.9), 0.8-1.0	0.6 (0.8), 0.3-0.9
I’m afraid I’m going to infect my family.	1.3 (0.7), 0.8-1.8	0.9 (1.0), 0.5-1.3	1.4 (1.0), 1.2-1.6	1.3 (1.0), 1.0-1.6
I have difficulty empathizing with patients’ suffering or connecting with their situation (emotional distancing and emotional anesthesia).	1.4 (1.1), 0.6-2.2	0.6 (1.1), 0.1-1.1	0.7 (1.9), 0.6-0.8	0.6 (0.9), 0.3-0.9
Total (scores range from 3 to 30)	*14.3 (5.5), 10.5-18.1*	*8.3 (6.6), 5.3-11.3*	*10.6 (6.4), 9.5-11.7*	*8.2 (5.0), 6.5-9.9*

^a^Individual scores range from 0 to 3 and italicized values represent statistical significance: *P*<.05.

^b^The first period was from March 25 to April 1, 2020; the second period was from April 1 to 19, 2020.

^c^This geographical area was most affected by COVID-19, with a higher mortality per 1000 inhabitants, and included Madrid, Catalonia, Basque Country, Aragon, Castile and Leon, and Valencia.

^d^This geographical area was least affected by COVID-19, with a lower mortality per 1000 inhabitants, and included Asturias, Canary Islands, La Rioja, Murcia, and Navarre.

### Criterion Validity

The EASE scale score was higher in the first period (March 25 to April 1, 2020) compared to the second period (April 1 to 19, 2020), in which the number of deaths per day decreased by half (mean 14.3, SD 5.5, 95% CI 10.5-18.1 vs mean 8.3, SD 6.6, 95% CI 5.3-11.3; *P*<.05) (see Table 3).

### Interpretability

The following categories based on score ranges were established: 0-9 points, good emotional adjustment; 10-14 points, emotional distress; 15-24 points, medium-high emotional overload; and ≥25 points, extreme acute stress. Most of the respondents were in the first range (115/228, 50.4%) and 28.9% (66/228) were in the second. Only 2.6% (6/228) were in the fourth bracket.

### Translation-Back-Translation

The Portuguese and English versions of the scale are shown in Multimedia Appendix 1.

### Availability of Data and Materials

Data and materials are available upon reasonable request.

## Discussion

### Principal Findings

The instrument designed and validated in this study has adequate metric properties and seems useful for professionals to become aware of their levels of stress while caring for COVID-19 patients during the pandemic. The average level of self-reported stress was between 9 and 11 points up to 30. This level of stress was measured during a period of unprecedented pressure while caring for COVID-19 patients. During this period, there were relevant organizational changes, uncertainty about the evolution of patients, a lack of personal protective equipment, and an increase in the number of professionals infected. It is expected that by decreasing the pressure caused by these situations, the emotional response of these health care professionals would increase as they become fully aware of their experience [3,16]. This scale has been used to identify and prevent such progression of stressful experiences among health professionals, the second victims of SARS-CoV-2 during the pandemic. The EASE scale has been linked to intervention measures to support the staff who are in direct contact with COVID-19 patients, using digital materials designed to improve psychological well-being [17]. This approach has been developed in several countries in addition to Spain [18,19].

This is particularly relevant because some current data in Spain using the EASE scale are suggesting that during the third wave, the level of distress reported by health care professionals is increasing up to 3 times, with 8.8% of respondents being classified into the fourth category of scores, probably as a result of accumulated fatigue and a feeling of starting over [5]. Therefore, caring for those who care has become a priority for public health strategies [20,21].

Stress, hypervigilance, fatigue, difficulty sleeping, inability to relax, and fear were common symptoms among health care professionals at the start of the pandemic [5]. The content validity analysis conducted in this study identified behaviors and responses related to these symptoms; the EASE scale items seek to assess the scale’s effect on those who provide care to COVID-19 patients. However, although mental symptoms have been identified, physical symptoms must also be considered when interventions to support them are being designed [20].

The EASE scale has sufficient sensitivity; the staff in contact with COVID-19 patients had higher levels of acute stress than other staff, as suggested in other studies [22]. When the EASE scale has been used in other studies, it was revealed that the levels of acute stress were higher in the absence of personal protective equipment, when the care pressure was greater, among professionals in critical or emergency units, and, as in this case, in those territories with a greater number of cases [23,24]. Since the current situation (ie, the third wave) seems different from the previous one, the scale’s utility in the next 3 to 6 months, after the worst of the current health crisis, should be checked.

In previous pandemics and in cases of natural disasters (earthquakes, tsunamis, etc), terrorist attacks with numerous victims, air or train crashes, and war conflicts, professionals have also experienced distress with consequences that have lasted a while [25]. COVID-19 is not the first pandemic in recent times. SARS-CoV in 2003 in China and Canada, MERS-CoV in 2012 in Saudi Arabia, and the Ebola outbreak in 2014 in several African countries, which reached Spain and other Organisation for Economic Co-operation and Development countries, impacted the well-being of health care professionals [26,27]. However, the magnitude of the health crisis caused by the COVID-19 pandemic has been more global and temporarily more widespread than in recent previous cases. Some signs of acute stress measured by the EASE scale are similar to all of these situations. However, some items are specific to an outbreak where the biological risk for professionals is present. In this sense, the EASE scale could be used in future outbreaks to check the level of acute stress of the health care workforce and to give them proportional support according to their needs in these conditions.

The rapid spread of SARS-CoV-2, coupled with the breakdown of the supply chain, lack of equipment, and uncertainty about how to deal with the treatment of COVID-19 patients, prompted the need for rapid responses to the pressing problems of the time. The EASE scale emerged at a time when the response capacity of professionals was under threat, as they were physically and mentally overwhelmed and many colleagues were becoming infected; in Spain, the number of professionals who were COVID-19 patients in the first wave were among the highest in the world.

The EASE scale includes four score brackets; each of them is linked to specific recommendations to address the psychological burden due to caring for COVID-19 patients [17]. Having a specific instrument such as the EASE scale to monitor acute stress during a pandemic can be beneficial for two reasons. First, it helps professionals become aware of their situation and could contribute to initiating self-help behaviors early on. Second, health care organizations have a “thermometer” with which to advise health care providers of when they should take a mandatory rest in order to benefit patients in their care, their colleagues, and themselves, before succumbing to the overload they are enduring.

Now, in this crisis, these same professionals should continue to care for patients with COVID-19, patients with sequelae from COVID-19, and all patients whose care processes have been interrupted during the acute phase of the pandemic. In this scenario, if rapid action is not taken, professionals’ capacity and, therefore, the quality and safety of patient care may be compromised.

This scenario may be a valuable opportunity to consolidate integrated care; now is the time to consider appropriate strategies to introduce measures that will increase health care professionals’ well-being at work and strengthen clinical leadership. In addition, it is time to commit to a model such as the Quadruple Aim of health care, which considers patient outcomes to be dependent on how caregivers are supported. The Quadruple Aim recognizes this focus within the context of the broader transformation required in our health care system toward high-value care. While the first three aims provide a rationale for a health system [28], the fourth aim becomes a foundational element for the other goals to be realized [29]. The key is the fourth aim: creating the conditions for the health care workforce to find joy and meaning in their work and improve the experience of providing care [30]. For this reason, it seems that “caring for the caregiver” [31] is necessary in the transformation of the health system, and having instruments to be able to monitor the effects of measures implemented can be very useful; at this point in time, this aim becomes “caring for the caregiver in times of pandemic” as a way to achieve optimal care for patients.

A limitation of this study is that it does not discriminate between professional categories, nor does it consider critical services separately during this crisis, such as critical care and resuscitation, internal medicine, pneumology, and infectious diseases. As well, these data are limited to Spain. Although a cultural and linguistic equivalence analysis of the scale was made, a measurement of the invariance among languages could be conducted in future studies. In the absence of a gold standard to assess criterion validity, in this study the ability of the scale to correctly classify respondents’ answers was considered.

In the forthcoming months, we can expect professionals to be affected during the outbreak as a consequence of stress overload [5], having seen their professional codes violated [3] due to insufficient resources to care for COVID-19 patients, contradictory instructions, or the interruption in the continuity of care of non-COVID-19 patients. Also, we can expect an increase of affective and anxiety reactions and symptoms, including, in some cases, posttraumatic stress among the professionals who saw their health and that of their loved ones threatened [32,33]. Continuing to monitor acute stress levels may be advisable in order to check on the effective recovery of health care professionals, which implies that the health of the population will continue to be adequately cared for.

Most probably, the initial impact due to the conditions in which the treatment of patients was carried out (ie, lack of equipment, lack of guidelines, overloading , and fear of contagion) is not related to the current experience of the health care providers. The recovery of professionals’ health, teamwork, and workers’ morale in health care organizations, particularly where the deceased include professionals from the team itself, will probably require specific and wide-ranging actions. Actions that promote a positive dynamic within the health care teams are more likely to be successful [5], for example, by reflecting on how they have acted, what has worked, and what could have been done differently [34-36].

### Conclusions

The EASE scale has been shown to exhibit adequate metric properties, such that it may be considered a reliable and valid scale. Its usefulness is two-fold: firstly, to help professionals become aware of their emotional overload and that it can be supported and, secondly, to measure the effect of this overload to avoid the progression toward more severe psychopathological conditions.

## Appendix

Multimedia Appendix 1 The Self-applied Acute Stress Scale (EASE).

## Abbreviations

CFA: confirmatory factor analysis
COSMIN: COnsensus-based Standards for the selection of health status Measurement INstruments
EASE: Self-applied Acute Stress Scale
MERS: Middle East respiratory syndrome

## References

[1] Huang C, Wang Y, Li X, Ren L, Zhao J, Hu Y, Zhang L, Fan G, Xu J, Gu X, Cheng Z, Yu T, Xia J, Wei Y, Wu W, Xie X, Yin W, Li H, Liu M, Xiao Y, Gao H, Guo L, Xie J, Wang G, Jiang R, Gao Z, Jin Q, Wang J, Cao B (2020). Clinical features of patients infected with 2019 novel coronavirus in Wuhan, China. Lancet.

[2] Zhu N, Zhang D, Wang W, Li X, Yang B, Song J, Zhao X, Huang B, Shi W, Lu R, Niu P, Zhan F, Ma X, Wang D, Xu W, Wu G, Gao GF, Tan W, China Novel Coronavirus Investigating and Research Team (2020). A novel coronavirus from patients with pneumonia in China, 2019. N Engl J Med.

[3] Greenberg N, Docherty M, Gnanapragasam S, Wessely S (2020). Managing mental health challenges faced by healthcare workers during COVID-19 pandemic. BMJ.

[4] Lai J, Ma S, Wang Y, Cai Z, Hu J, Wei N, Wu J, Du H, Chen T, Li R, Tan H, Kang L, Yao L, Huang M, Wang H, Wang G, Liu Z, Hu S (2020). Factors associated with mental health outcomes among health care workers exposed to coronavirus disease 2019. JAMA Netw Open.

[5] Vanhaecht K, Seys D, Bruyneel L, Cox B, Kaesemans G, Cloet M, Van Den Broeck K, Cools O, De Witte A, Lowet K, Hellings J, Bilsen J, Lemmens G, Claes S (2021). COVID-19 is having a destructive impact on health-care workers' mental well-being. Int J Qual Health Care.

[6] Kisely S, Warren N, McMahon L, Dalais C, Henry I, Siskind D (2020). Occurrence, prevention, and management of the psychological effects of emerging virus outbreaks on healthcare workers: Rapid review and meta-analysis. BMJ.

[7] (2020). Coronavirus Disease 2019 (COVID-19) in the EU/EEA and the UK– ninth update.

[8] Liu C, Yang Y, Zhang X, Xu X, Dou Q, Zhang W, Cheng ASK (2020). The prevalence and influencing factors in anxiety in medical workers fighting COVID-19 in China: A cross-sectional survey. Epidemiol Infect.

[9] Cai H, Tu B, Ma J, Chen L, Fu L, Jiang Y, Zhuang Q (2020). Psychological impact and coping strategies of frontline medical staff in Hunan between January and March 2020 during the outbreak of coronavirus disease 2019 (COVID‑19) in Hubei, China. Med Sci Monit.

[10] Ibáñez-Vizoso JE, Alberdi-Páramo Í, Díaz-Marsá M (2020). International mental health perspectives on the novel coronavirus SARS-CoV-2 pandemic. Rev Psiquiatr Salud Ment.

[11] Galbraith N, Boyda D, McFeeters D, Hassan T (2020). The mental health of doctors during the COVID-19 pandemic. BJPsych Bull.

[12] Boateng GO, Neilands TB, Frongillo EA, Melgar-Quiñonez HR, Young SL (2018). Best practices for developing and validating scales for health, social, and behavioral research: A primer. Front Public Health.

[13] Chan EKH, Zumbo BD, Chan EKH (2014). Standards and guidelines for validation practices: Development and evaluation of measurement instruments. Validity and Validation in Social, Behavioral, and Health Sciences. Social Indicators Research Series, vol 54.

[14] Mokkink LB, Terwee CB, Patrick DL, Alonso J, Stratford PW, Knol DL, Bouter LM, de Vet HCW (2010). The COSMIN checklist for assessing the methodological quality of studies on measurement properties of health status measurement instruments: An international Delphi study. Qual Life Res.

[15] Garfin DR, Thompson RR, Holman EA (2018). Acute stress and subsequent health outcomes: A systematic review. J Psychosom Res.

[16] Borges LM, Bahraini NH, Holliman BD, Gissen MR, Lawson WC, Barnes SM (2020). Veterans' perspectives on discussing moral injury in the context of evidence-based psychotherapies for PTSD and other VA treatment. J Clin Psychol.

[17] Mira JJ, Vicente MA, Lopez-Pineda A, Carrillo I, Guilabert M, Fernández C, Pérez-Jover V, Martin Delgado J, Pérez-Pérez P, Cobos Vargas A, Astier-Peña MP, Martínez-García OB, Marco-Gómez B, Abad Bouzán C (2020). Preventing and addressing the stress reactions of health care workers caring for patients with COVID-19: Development of a digital platform (Be + Against COVID). JMIR Mhealth Uhealth.

[18] Strametz R, Raspe M, Ettl B, Huf W, Pitz A (2020). Recommended actions: Reinforcing clinicians' resilience and supporting second victims during the COVID-19 pandemic to maintain capacity in the healthcare system [Article in German]. Zentralbl Arbeitsmed Arbeitsschutz Ergon.

[19] Blake H, Bermingham F, Johnson G, Tabner A (2020). Mitigating the psychological impact of COVID-19 on healthcare workers: A digital learning package. Int J Environ Res Public Health.

[20] Salazar de Pablo G, Vaquerizo-Serrano J, Catalan A, Arango C, Moreno C, Ferre F, Shin JI, Sullivan S, Brondino N, Solmi M, Fusar-Poli P (2020). Impact of coronavirus syndromes on physical and mental health of health care workers: Systematic review and meta-analysis. J Affect Disord.

[21] Morgantini L, Naha U, Wang H, Francavilla S, Acar Ö, Flores J, Crivellaro S, Moreira D, Abern M, Eklund M, Vigneswaran HT, Weine SM (2020). Factors contributing to healthcare professional burnout during the COVID-19 pandemic: A rapid turnaround global survey. PLoS One.

[22] Kisely S, Warren N, McMahon L, Dalais C, Henry I, Siskind D (2020). Occurrence, prevention, and management of the psychological effects of emerging virus outbreaks on healthcare workers: Rapid review and meta-analysis. BMJ.

[23] Mira JJ, Carrillo I, Guilabert M, Mula A, Martin-Delgado J, Pérez-Jover MV, Vicente MA, Fernández C, SARS-CoV-2 Second Victim Study Group (2020). Acute stress of the healthcare workforce during the COVID-19 pandemic evolution: A cross-sectional study in Spain. BMJ Open.

[24] Martin-Delgado J, Viteri E, Mula A, Serpa P, Pacheco G, Prada D, Campos de Andrade Lourenção D, Campos Pavan Baptista P, Ramirez G, Mira JJ (2020). Availability of personal protective equipment and diagnostic and treatment facilities for healthcare workers involved in COVID-19 care: A cross-sectional study in Brazil, Colombia, and Ecuador. PLoS One.

[25] Mattei A, Fiasca F, Mazzei M, Necozione S, Bianchini V (2017). Stress and burnout in health-care workers after the 2009 L'Aquila earthquake: A cross-sectional observational study. Front Psychiatry.

[26] Khalid I, Khalid TJ, Qabajah MR, Barnard AG, Qushmaq IA (2016). Healthcare workers emotions, perceived stressors and coping strategies during a MERS-CoV outbreak. Clin Med Res.

[27] Lehmann M, Bruenahl CA, Löwe B, Addo MM, Schmiedel S, Lohse AW, Schramm C (2015). Ebola and psychological stress of health care professionals. Emerg Infect Dis.

[28] Berwick DM, Nolan TW, Whittington J (2008). The Triple Aim: Care, health, and cost. Health Aff (Millwood).

[29] Sikka R, Morath JM, Leape L (2015). The Quadruple Aim: Care, health, cost and meaning in work. BMJ Qual Saf.

[30] Bodenheimer T, Sinsky C (2014). From Triple to Quadruple Aim: Care of the patient requires care of the provider. Ann Fam Med.

[31] Karb R, Natsui S (2019). Reflections: Addressing the Quadruple Aim in social emergency medicine. Ann Emerg Med.

[32] Chen Q, Liang M, Li Y, Guo J, Fei D, Wang L, He L, Sheng C, Cai Y, Li X, Wang J, Zhang Z (2020). Mental health care for medical staff in China during the COVID-19 outbreak. Lancet Psychiatry.

[33] Kang L, Ma S, Chen M, Yang J, Wang Y, Li R, Yao L, Bai H, Cai Z, Xiang Yang B, Hu S, Zhang K, Wang G, Ma C, Liu Z (2020). Impact on mental health and perceptions of psychological care among medical and nursing staff in Wuhan during the 2019 novel coronavirus disease outbreak: A cross-sectional study. Brain Behav Immun.

[34] Traylor AM, Tannenbaum SI, Thomas EJ, Salas E (2021). Helping healthcare teams save lives during COVID-19: Insights and countermeasures from team science. Am Psychol.

[35] Janssen M, van der Voort H (2020). Agile and adaptive governance in crisis response: Lessons from the COVID-19 pandemic. Int J Inf Manage.

[36] Schippers M (2020). Optimizing decision-making processes in times of COVID-19: Using reflexivity to counteract information processing failures. ERIM Report Series reference forthcoming. SSRN.

