# Supplementary material for: An Acute Stress Scale for Health Care Professionals Caring for Patients With COVID-19: Validation Study
Source: JMIR Form Res. 2021 Mar 9;5(3):e27107. doi: 10.2196/27107 (PMC7945973; doi:10.2196/27107)
Supplement: Multimedia Appendix 1 [file formative_v5i3e27107_app1.docx]

Supplementary files: EASE Scale in English Version, Spanish (Spain) Version, Spanish (Latin American) Version, and Brazilian Portuguese version.

| *Please answer the following questions according to the thoughts, emotions, sensations and actions you are experiencing during these days of crisis* | | | | |
| --- | --- | --- | --- | --- |
|  | It's not  happening to me | It happens to me  in concrete situations | It often happens to me | I'm like this all the time |
| I can't help but think of recent critical situations. I can't get out of work | **□** | **□** | **□** | **□** |
| I have completely lost the taste for things that gave me peace of mind | **□** | **□** | **□** | **□** |
| I keep my distance, I resent dealing with people, I'm irascible even at home | **□** | **□** | **□** | **□** |
| I feel that I am neglecting many people who need my help | **□** | **□** | **□** | **□** |
| I have difficulty thinking and making decisions, I have many doubts, I have entered a kind of emotional blockage | **□** | **□** | **□** | **□** |
| I feel intense physiological reactions (shocks, sweating, dizziness, shortness of breath, insomnia, etc.) related to the current crisis situation | **□** | **□** | **□** | **□** |
| I feel on permanent alert. I believe that my reactions now put other patients, my colleagues or myself at risk | **□** | **□** | **□** | **□** |
| Worrying about not getting sick causes me a strain that's hard to bear | **□** | **□** | **□** | **□** |
| I'm afraid I'm going to infect my family | **□** | **□** | **□** | **□** |
| I have difficulty empathizing with patients' suffering or connecting with their situation (emotional distancing, emotional anesthesia) | **□** | **□** | **□** | **□** |

| *Contesta por favor a las siguientes preguntas acorde a los pensamientos, emociones, sensaciones y acciones que has experimentado durante estos días de crisis.* | | | | |
| --- | --- | --- | --- | --- |
|  | No me está sucediendo | Me pasa en situaciones concretas | Me pasa a menudo | Estoy así continuamente |
| No puedo evitar que me vengan a la cabeza situaciones críticas recientes. No logro desconectar del trabajo. | **□** | **□** | **□** | **□** |
| He perdido por completo el gusto por las cosas que antes me producían tranquilidad o bienestar. | **□** | **□** | **□** | **□** |
| Me mantengo distante, me molesta el trato con la gente, estoy irascible incluso en casa. | **□** | **□** | **□** | **□** |
| Siento que estoy descuidando a muchas personas que requieren de mi ayuda. | **□** | **□** | **□** | **□** |
| Tengo dificultades para pensar y tomar decisiones, tengo muchas dudas, he entrado en una especie de estado de bloqueo emocional. | **□** | **□** | **□** | **□** |
| Siento reacciones fisiológicas intensas (sobresaltos, sudoración, mareos, falta de respiración, insomnio, etc.) relacionadas con la situación actual de crisis. | **□** | **□** | **□** | **□** |
| Me siento en estado de alerta permanente. Creo que mis reacciones ahora ponen en riesgo a otros pacientes, a mis colegas o a mí mismo/a. | **□** | **□** | **□** | **□** |
| La preocupación por no caer enfermo me provoca una tensión difícil de soportar. | **□** | **□** | **□** | **□** |
| Tengo miedo por si voy a contagiar a mi familia. | **□** | **□** | **□** | **□** |
| Tengo dificultades para empatizar con el sufrimiento de los pacientes o conectar con su situación (distanciamiento emocional, anestesia afectiva). | **□** | **□** | **□** | **□** |

| *Por favor, contesta a las siguientes preguntas en función de los pensamientos, emociones, sensaciones y acciones que estás experimentando durante estos días de crisis:* | | | | |
| --- | --- | --- | --- | --- |
|  | No me ocurre | Me ocurre en situaciones concretas | Me ocurre casi siempre | Estoy así todo el tiempo |
| No puedo evitar que me vengan a la cabeza situaciones críticas recientes. No logro desconectar del trabajo una vez que llego a casa. | **□** | **□** | **□** | **□** |
| He perdido por completo el gusto por las cosas que antes me producían tranquilidad o bienestar. | **□** | **□** | **□** | **□** |
| Me mantengo distante, me molesta el trato con la gente, estoy irritable incluso en casa. | **□** | **□** | **□** | **□** |
| Siento que estoy descuidando a muchas personas que requieren de mi ayuda. | **□** | **□** | **□** | **□** |
| Tengo dificultades para pensar y tomar decisiones, he entrado en una especie de estado de bloqueo emocional. | **□** | **□** | **□** | **□** |
| Siento reacciones fisiológicas intensas (sobresaltos, sudoración, mareos, falta de respiración, insomnio, etc.) relacionadas con la crisis actual. | **□** | **□** | **□** | **□** |
| Me siento en estado de alerta permanente. Creo que mis reacciones ahora ponen en riesgo a otros pacientes, a mis colegas o a mí mismo/a. | **□** | **□** | **□** | **□** |
| La preocupación por no enfermar me provoca una tensión difícil de soportar. | **□** | **□** | **□** | **□** |
| Tengo miedo de contagiar a mi familia. | **□** | **□** | **□** | **□** |
| Tengo dificultades para empatizar con el sufrimiento de los pacientes o conectar con su situación (distanciamiento emocional, anestesia afectiva). | **□** | **□** | **□** | **□** |

| *Responda às perguntas abaixo com base nos pensamentos, emoções, sentimentos e ações que você está enfrentando durante esses dias de crise.* | | | | |
| --- | --- | --- | --- | --- |
|  | Isso não está acontecendo comigo | Isso acontece comigo em situações concretas | Isso acontece comigo muitas vezes | Eu sou assim continuamente |
| Não consigo impedir que situações críticas recentes surjam na minha cabeça. Não consigo me desconectar do trabalho. | **□** | **□** | **□** | **□** |
| Perdi completamente o gosto pelas coisas que antes me davam tranquilidade ou bem-estar. | **□** | **□** | **□** | **□** |
| Me mantenho distante, interagir com as pessoas me incomoda, sou irascível até em casa | **□** | **□** | **□** | **□** |
| Sinto que estou negligenciando muitas pessoas que precisam da minha ajuda | **□** | **□** | **□** | **□** |
| Tenho dificuldades para pensar e tomar decisões, tenho muitas dúvidas, entrei em uma espécie de estado de bloqueio emocional. | **□** | **□** | **□** | **□** |
| Sinto reações fisiológicas intensas (sobressaltos, sudorese, tonturas, falta de ar, insônia etc.) relacionadas à atual situação de crise. | **□** | **□** | **□** | **□** |
| Sinto-me constantemente em alerta. Acho que minhas reações agora colocam em risco os pacientes, meus colegas ou eu mesmo. | **□** | **□** | **□** | **□** |
| A preocupação de não adoecer causa uma tensão difícil de suportar. | **□** | **□** | **□** | **□** |
| Estou com medo, caso eu vá infectar minha família | **□** | **□** | **□** | **□** |
| Tenho dificuldade de simpatizar com o sofrimento dos pacientes ou de me conectar com a situação deles (distanciamento emocional, anestesia afetiva). | **□** | **□** | **□** | **□** |
